# The Impacts of Flattening Fiscal Reform on Health Expenditure in China

**DOI:** 10.3389/fpubh.2021.614915

**Published:** 2021-04-26

**Authors:** Jun Hao, Chengxiang Tang, Junli Zhu, Jiayi Jiang

**Affiliations:** ^1^School of Public Health, Capital Medical University, Beijing, China; ^2^Medical Research and Biometrics Center, National Center for Cardiovascular Diseases, Fuwai Hospital, Chinese Academy of Medical Sciences, Peking Union Medical College, Beijing, China; ^3^School of Public Administration, Guangzhou University, Guangzhou, China; ^4^China Center for Health Economic Research, Peking University, Beijing, China

**Keywords:** fiscal reform, health expenditure, public finance, difference-in-difference, Province-Managing-County

## Abstract

**Introduction:** A number of provinces have implemented a fiscal reform of flattening government since the first decade of this century in China. This study aims to quantitatively analyze the influences of this government fiscal reform on county-level health expenditure. We also bring forward policy suggestions for improving county-level fiscal system and healthcare delivery.

**Methods:** We collected a novel longitudinal county-level data from 2003 to 2010, including counties' socioeconomic data, fiscal revenue, and health expenditure. Jilin Province, Hebei Province, and Anhui Province were selected as representative samples for this policy evaluation. The study employed a time-varying difference-in-difference model specification to investigate the impacts of flattening fiscal reform on health expenditure.

**Results:** The analyses find that the fiscal system reforms of the three provinces have a significantly positive impact on the health expenditure of county-level governments. However, we find no policy effects on the proportion of health expenditure to fiscal expenditure of county-level governments. The estimation results are robust after controlling several background variables.

**Conclusion:** The results yield important policy insights that public finance and its reform significantly impacts health expenditures in China. The government may still need to strengthen the transfer payment system to guarantee the social welfare provision in healthcare.

## Introduction

Universal Health Coverage (UHC) has become a health strategy goal of many countries in the world. UHC requires that everyone has access to the services they need within the health system, where these services are of adequate quality to be effective, and get universal financial protection in the costs of using these services. It is the governments' responsibilities to provide domestic resources to the maximum extent possible in order to fulfill their commitments to the health and other human rights of their citizens (1, 2). China's total health expenditure consists of three parts: government budget health expenditure, social insurance expenditure, and individual health expenditure. Government budget health expenditure refers to the financial allocation of the central and local governments for healthcare, in which the health expenditure defined in this study specifically refers to county-level governments' health expenditure. In 1997, the “Decision on Health Reform and Development” issued by the Central Committee of the Communist Party of China and the State Council clearly stated that the governments take important responsibility for the development of health services, and that the government health expenditure should be increased with the development of the economy and the growth rate should not be lower than that of fiscal expenditure. “Plan of Health China 2030” released in 2016 also emphasized adjusting and optimizing the structure of fiscal expenditure and increasing government expenditure in health sectors. China's total health expenditure report for 2019 showed that the total health expenditure accounted for 6.6% of the gross domestic product (GDP), of which the government health expenditure accounted for 26.7% of the total health expenditure. Although the governments have increased health expenditure since severe acute respiratory syndrome (SARS) in 2003, the governments' role in health expenditure is extremely weak when compared with other countries, especially Organization for Economic Cooperation and Development (OECD) countries (3). The central and local governments in China have been inactive in providing healthcare for citizens, and China's health sectors have been mainly financed by the private sectors (3).

The financial capacity of county governments is crucial to ensure adequate health services for their residents. Since 1994, China has implemented a hierarchical tax-sharing budget management system (referred to as the “tax-sharing system”) clarifying the division of fiscal revenue and responsibilities between the central and local governments. China has gradually become one of the countries with a high degree of fiscal decentralization in the world since then (4). The central and provincial governments are responsible for the broader policy and strategic design and investment in the larger health infrastructure, whereas the county-level governments have practical responsibilities for implementing health programs or services (5). Data showed that more than 98% of government health expenditures in China mainly rely on local government finances (6). Especially, the county-level government plays an important role in the construction of the county-level healthcare system and undertakes important tasks, such as ensuring the equalization of basic public health services.

In China, fiscal revenue was mainly concentrated in higher-level governments, namely, the central, provincial, and prefectural governments, whereas county-level and township-level financial resources were very weak. There was a mismatch of financial power and administrative power between cities and counties. China established a hierarchical structure of governance in 1982, which consists of five layers of government—from the highest to the lowest, they are as follows: the central level, the province or municipality level, the prefecture or city level (hereafter city-level), the county level, and the township level (7). However, it is evident that the five-level administrative system has caused many problems with the rapid development of China's economy and society since the 1980s and the early 2000s, one of which is that city-level governments allocated more financial resources to cities, forming a situation of a “city-scraping county” (8). Besides, a reform of the tax-sharing fiscal system mentioned above was carried out in China in 1994, which initially clarified the fiscal and taxation relations between the central and provincial governments, whereas the relations below the province-level government were still not illuminated. As a result, fiscal revenue was mainly concentrated in the central, provincial, and prefectural governments, whereas financial resources at the county level and township level were very weak. This resulted in the mismatch of financial power and administrative power between cities and counties (9). Data showed that the proportion of county and township fiscal expenditures in China's fiscal expenditures has increased by year since 2000, and that it has become the most responsible government for fiscal expenditures since 2003. At the same time, the county and township fiscal revenues only accounted for 20% of the national fiscal revenue (10). The county and township financial system is so poor that many county governments suffered from insufficient allocation of basic public services.

Given that situation, all sectors of society began to actively explore new management systems. A fiscal decentralization reform was implemented in China's local governments, which eliminated the city-level government as the intermediate layer between the province and the county (flattening fiscal reform). In 1992, Zhejiang Province creatively put forward a flattening fiscal reform that reduced the five-level hierarchical structure to a four-level system—central level, province or municipality level, county level, and township level. After that, Zhejiang Province carried out five rounds of power expansion reform, and its county economy developed rapidly. Compared with the economically developed coastal provinces, such as Jiangsu, Guangdong, Shandong, and Fujian, Zhejiang's county economy developed to a higher and more balanced level (11). As a new exploration of fiscal decentralization mode, the flattening fiscal system has achieved remarkable results in Zhejiang Province, prompting the Ministry of Finance to carry out the flattening fiscal reform nationwide since 2002, known as the pilot work of Province-Managing-County (PMC) reform (12, 13).

The PMC was a reform to flatten governmental hierarchical structure, which made huge innovations in the expansion of economic rights of the grassroots government and the deepening of fiscal decentralization. This reform enabled county governments directly governed by the provincial government *via* the public finance transfer payment, fund dispatching, financial settlements, and work deployment, no longer subject to the prefectural finance. The purpose of the reform is to improve local economic development, augment county-level finance capacity, and equalize the supply of public services across counties (14). The reform was first piloted in counties in central and northern China, such as Anhui Province, Hebei Province, Liaoning Province, and Jilin Province. In 2006, the reform was extended to Jiangsu Province, Shaanxi Province, Sichuan Province, Gansu Province, and Qinghai Province in eastern and western China. The Ministry of Finance proposed that the PMC reform should be promoted across all provinces, except minority autonomous regions by 2012 in the Opinions on Promoting Province-Managing-County Reform [No. 78 (2009) of The Ministry of Finance). By the end of 2012, 1,099 counties from 24 provinces, representing ~56% of all counties across the country, had implemented the PMC reform (15).

Until now, there has been little research on the impact of the PMC reform on government health expenditure, especially from the perspective of fiscal reform, so the impact of the PMC reform on county-level government health expenditure has been unclear yet. The analysis of the factors that influence China's local government health expenditure and health service provision from a new perspective, namely, fiscal system reform, will provide a powerful support for better promoting UHC policies, such as equalization of basic public health services. Therefore, it will be necessary and interesting to study government health expenditure in these counties from the perspective of the fiscal system. This research will focus on the impact of the PMC reform on county-level government health expenditure, employing a panel data set of county-level governments in Hebei Province, Anhui Province, and Jilin Province in China during 2003–2010.

## Literature Review

The previous studies have focused on the factors of influencing government health expenditure from multiple perspectives, such as macro-economic variables. Cantarero found that the aging population was the most important determinant of the regional health expenditure in Spain (16). Rahman showed that per capita income and literacy rate had an important impact on per capita health expenditure in India from 1971 to 1991 (17). Behera and Dash studied the long-run effects of GDP and tax revenue on public health expenditure in 16 major states of India during 1980–2014 (18). He analyzed China's 2000–2011 provincial panel data and found that per capita GDP had a significant impact on the government health expenditure, whereas population size, population structure, and urbanization had no effects (19). Lu & Wang found that economic growth, aging degree, and other factors positively affected per capita public health expenditure by an empirical research on provincial panel data of China from 2002 to 2006 (20).

Early scholars explained fiscal decentralization from the low-level government to understand the preferences of residents in the jurisdiction (21). Recently, some scholars believed that fiscal decentralization could encourage local officials to improve the welfare of residents in the jurisdiction (22, 23). Most studies have shown that fiscal decentralization benefited public health and improved public outcomes, such as widening childhood immunization coverage and reducing infant mortality rates (24–27). Since the reform of the tax-sharing system in 1994, China has gradually become one of the countries with a high degree of fiscal decentralization in the world (4). Several studies on the impact of fiscal decentralization in China have emerged, but these studies have not reached a consistent conclusion as international evidences and there are only a few articles on the impact on health. Peng & Tang proved that fiscal decentralization promoted the supply of health services (28), whereas Sun and Jin gave the evidence that fiscal decentralization did not reduce infant mortality by using provincial panel data (29).

Because the PMC reform in China involved a sudden change in vertical governmental structure and was implemented only in certain localities at a certain point in time, it is a quasi-natural experiment, providing an opportunity to evaluate the impact of fiscal decentralization on public expenditures of the local governments in China. There have been a growing number of papers in recent years on the PMC reform, especially in Chinese. A study suggested that the PMC reform may have a significant impact on the scale and composition of government expenditures, and that it increased the productive expenditure of a county government (30, 31), but the evidence for the impact on welfare expenditure has been still inconclusive. Some studies showed that the PMC reform was found to increase local welfare expenditure, although it did not change the motivation of the local government to allocate more funds to productive public services rather than to civil public services (14, 30). Another research insisted that the PMC reform decreased local welfare expenditure as more resources were available to increase productive expenditure (31, 32). Although the local government in China has taken important responsibilities for health services, there is no study that evaluates the effects of the PMC reform on the health expenditure of local governments. In one related study, the authors only chose health expenditure as one kind of county governments' welfare expenditure and found that the PMC reform has a negative effect on the proportion of health expenditure to fiscal expenditure (32).

## Methods and Data

### Study Data

This study uses data and documents related to the PMC fiscal reform from provincial government websites and the *China Statistical Yearbooks Database* (CSYD). Considering the availability of data and the gap between the economic development level of the western provinces and the central and eastern provinces in China, the western provinces have not been included.

**Figure 1:**
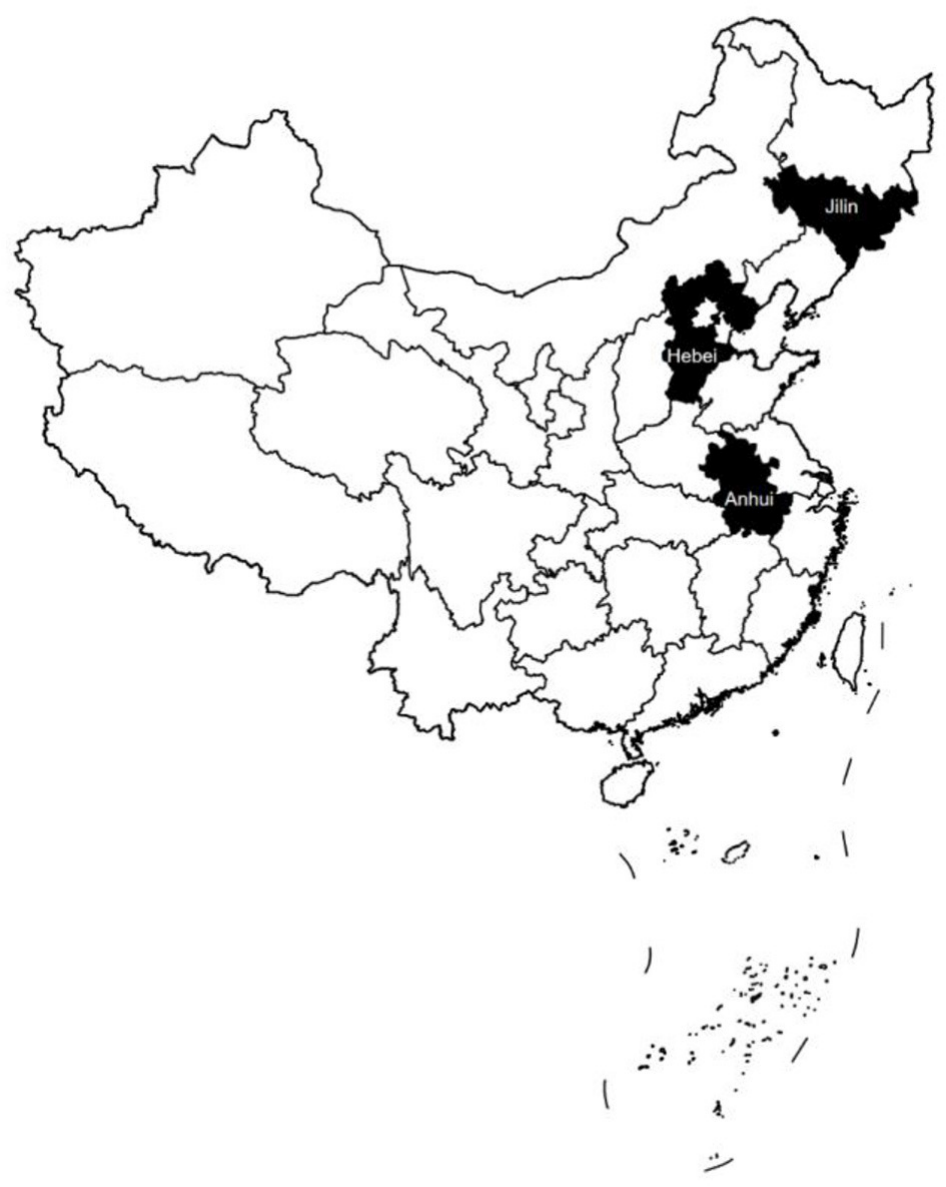
Three provinces that implemented fiscal reform after 2005.

Taking into account the representativeness of the sample and the availability of the data, the study took counties (county-level cities) in Jilin Province (northeast region), Hebei Province (east region), and Anhui Province (central region) as the research objects (see Figure 1). In addition, the counties (county-level cities) that have undergone county-to-district or county-to-city administrative territorial entity adjustment during the sample period have been excluded. Therefore, the final sample set of this study was 237 counties (including 190 counties and 47 county-level cities) in Jilin Province from 2001 to 2010, Hebei Province from 2003 to 2010, and Anhui Province from 2003 to 2010, with a total of 1,976 observations. Because of the different reform time points of the three provinces, it was necessary to construct a multi-period difference-in-difference (DID) model to study the impact of the PMC fiscal reform in the three provinces on the county-level health expenditure, which requires the balanced panel data. Therefore, when analyzing the policy effect of the PMC fiscal reform in these three provinces, we treated the sample period as 2003–2010, 237 counties (county-level cities) altogether, and 1,896 observations. In January 2005, 22 counties in Hebei Province (16%, *n* = 136) were involved in the PMC fiscal reform, along with 32 counties in Jilin Province (80%, *n* = 40) in June 2005 and 57 counties in Anhui Province (93%, *n* = 61) in January 2004.

By consulting the relevant policy documents of the PMC fiscal reform in each province, we were able to see the progress of the reform and manually input the data, such as the reform time point. Social and economic data like county population, GDP, and financial data, such as fiscal revenue and expenditure, were collected by consulting the statistical yearbook and fiscal yearbook. Some missing data were obtained from city-level statistical yearbooks. Data processing and statistical analysis were performed using Stata 15.1.

### Dependent Variable

In this study, the health expenditure (healthexp) and its proportion to fiscal expenditure (healthperc) were taken as dependent variables. The proportion of health expenditure to fiscal expenditure (healthperc) can be used to measure the government's investment in healthcare.

### Independent Variable

In this study, the independent variable was the dummy variable of the PMC fiscal reform. One represents the value of a county (county-level cities) in the year of the fiscal reform and after the reform, and 0 represents the value of the year before the reform. Based on previous literature, this study controlled for variables that may have an impact on health expenditure. First, the level of economic development will affect the government's revenue-raising capacity, which has an impact on health expenditure, so we controlled for variables that reflect the level of the economic development—per capita gross domestic product (avgGDP) and second industry output (secindustry). Second, the population scale will affect government operation in every aspect, so the population of each county was included (pop). In order to reduce the influence of heteroscedasticity, we made log transformations for the above variables into lnavgGDP, lnpop, and lnsecindustry. Self-financing capacity (capacity) refers to the percentage of the fiscal revenue in fiscal expenditure. The self-financing capacity of the local government will affect the structure of fiscal expenditure directly, so it was included in the model. Since both counties and county-level cities have carried out PMC reforms, county-level cities have already been coordinated by provinces. Therefore, we added a dummy variable (county2) into the model in order to control the impact of different administrative units' division −1 for county-level city and 0 for county.

### Identifying Strategy

The DID model is generally employed to evaluate the effect of policy implementation (33, 34), and the actual effect of policy variable is obtained through fixed-effect analysis of panel data. There have been many studies using the DID model to evaluate the effect of policy implementation, such as many empirical analyses of the policy effect of the PMC reform. Besides, the treatment structure in our study is characterized by varying policy start dates; thus, we use the following time-varying DID model specification to estimate the effect of the PMC reform on county-level health expenditure:

(1) $$\begin{array}{r} {Healthexpend}_{it}\;=\;\beta_{1}{Treatment}_{it}\;+\;{year}_{t}+C_{i}\;+\;X_{it}\;+\;\varepsilon_{it}. \end{array}$$

**Figure 2:**
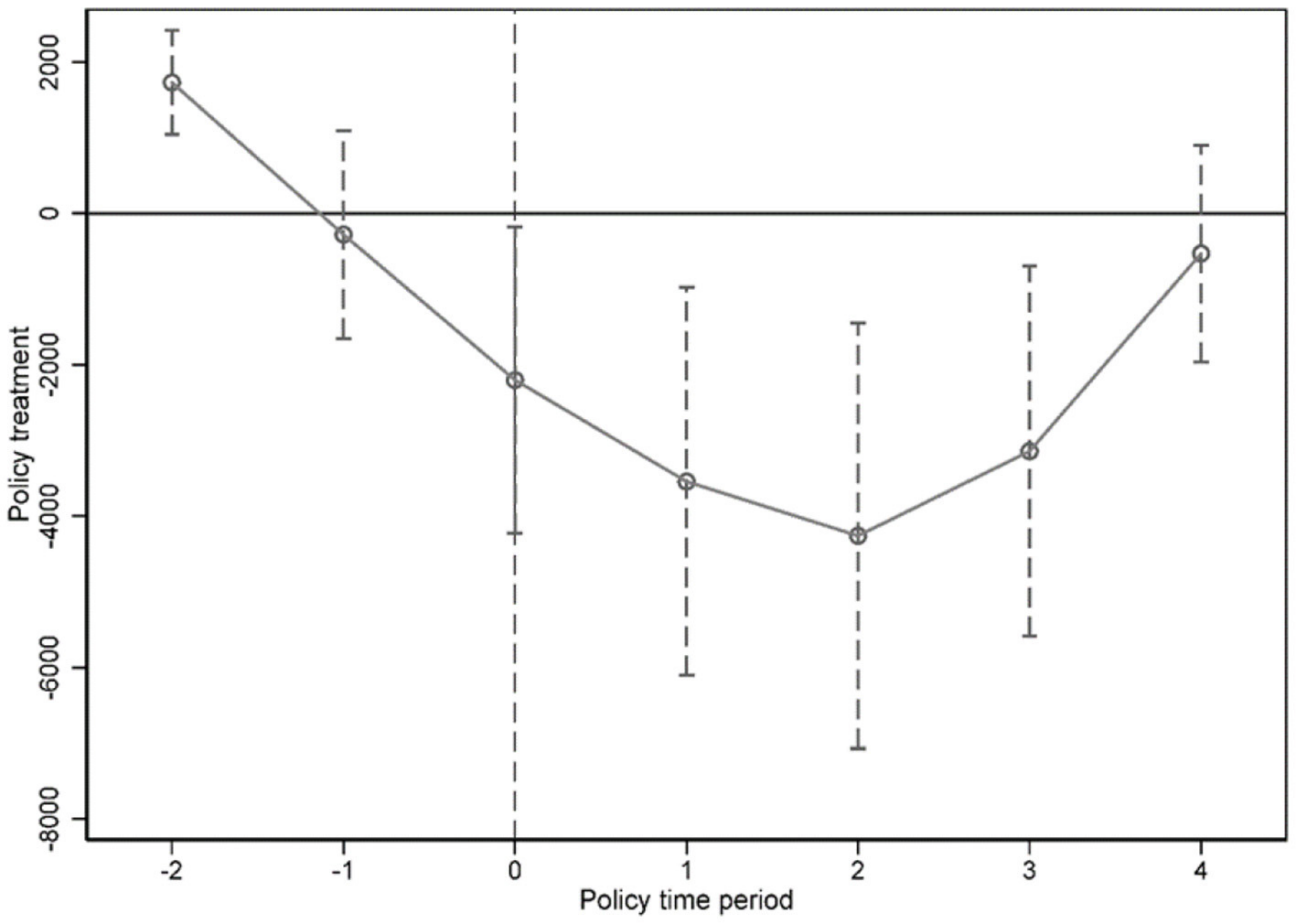
Common trend test before and after fiscal reform.

For any observation county *i* in year *t, Treatment*_*it*_ is a dummy variable equal to 1 for the year that a county started to introduce the PMC fiscal reform and 0 for the years after. The coefficient of interest β_1_ captures the average change in the health expenditure during the process of fiscal reform. In this two-way fixed-effects model, a set of year dummies *year*_*t*_ and county dummies *C*_*i*_ capture the differences fixed over years and across counties. Other control variables that change across counties and over time, *X*_*it*_, include the log of population, the log of per capita GDP, and the log of second industry output, which may affect the health expenditure. We have reported robust standard errors clustered at the county level. For this identification strategy, we tested for assumption of common trend between the treatment group and the control group (see Figure 2).

## Results

Table 1 shows the descriptive statistics of selected variables. Relative to the treatment group, the control group had lower health expenditure; however, the proportion of health expenditure to fiscal expenditure showed little difference between the two groups in the outcome variables. In the control variables, the mean values of self-financing capacity, per capita GDP, population, and second industry output of the treatment group were higher than those of the control group.

**Table 1:** Descriptive statistics of outcome and control variables in the DID model.

**Variables**	**Treated**					**Control**				
	**Obs**	**Mean**	**Std. Dev**.	**Min**	**Max**	**Obs**	**Mean**	**Std. Dev**.	**Min**	**Max**
Healthexp	886	6,486.01	6,742.29	342	36,267	754	3,329.66	4,043.23	168	25,428
Healthperc	886	0.06	0.03	0.01	0.17	754	0.06	0.03	0.01	0.16
Capacity	888	0.31	0.16	0.06	2.05	1,008	0.29	0.14	0.05	1.31
AvgGDP	888	13,019.81	10,580.55	1,712	97,690.98	1,008	13,784.89	9,432.89	2,619.58	84,205.31
County2	888	0.28	0.45	0	1	1,008	0.13	0.33	0	1
Pop	888	66.26	39.34	8.32	220	1,008	39.74	16.90	11	121
Secindustry	888	331,480	431,992.8	10,300	4,744,940	1,008	271,758.1	248,796.5	8,716	2,097,890

*Healthexp is the health expenditure of each county; Healthperc is the portion of health expenditure to fiscal expenditure; capacity is the self-financing capacity, namely, the proportion of county fiscal revenue to fiscal expenditure; AvgGDP is the per capita GDP; County2 is a dummy variable, “1” for county-level city and “0” for county; Pop is the population of each county; Secindustry is the second industry output*.

Table 2 presents the regression results for health expenditure and its proportion to fiscal expenditure. All columns consist of the county-level control including self-financing capacity, per capita GDP, population, and second industry output. Besides, a set of year fixed effects and county fixed effects were included. Standard errors shown in parentheses were clustered at the county level. It was found that the PMC reform of the three provinces had a significantly positive effect on the health expenditure of county-level governments (β_1_ = 2,004.9, *p* < 0.001), whereas we found no effects on the proportion of health expenditure to fiscal expenditure of county-level governments (β_1_ = 0.00, *p* > 0.05).

**Table 2:** Main results from the DID model.

**Variables**	**Healthexp**	**Healthperc**
Treatment	2,004.9^**^ (6.99)	0.00 (0.59)
lnavgGDP	2,072.5 (1.94)	−0.02^**^ (−5.56)
Lnpop	8,915.4^*^ (2.65)	0.07^**^ (3.63)
Capacity	−3,067.9^*^ (−3.18)	−0.01 (−0.47)
Lnsecindustry	703.0 (1.24)	0.00 (1.37)
_cons	−58,112.4^**^ (−3.78)	−0.03 (−0.31)
N	1,640	1,640

*t statistics are in parentheses*.

* *p < 0.01*,

** *p < 0.001*.

To guarantee reliability, we did a robustness inspection to test the reliability of the conclusion. As is shown in Table 3, only the treatment variable was added in column (1). In column (2), self-financing capacity, per capita GDP, population, and second industry output were added. On the basis of column (2), we added a control variable for a dummy variable indicating whether the county is a county-level city. Columns (4), (5), and (6) were hierarchical regression. Column (4) is the regression result of counties with population higher than 52.16; column (5) is the regression result of counties with self-financing capacity lower than 0.30, and the final column is the result of counties with per capita GDP higher than 13,426.7. Table 3 shows that the PMC fiscal reform had a significant impact on county-level health expenditure but no effects on the proportion of health expenditure to fiscal expenditure. Table 3 shows that for health expenditure, the estimated coefficients are statistically significant in the sample period.

**Table 3:** Robustness results from different specifications.

**Variables**	**(1)**	**(2)**	**(3)**	**(4)**	**(5)**	**(6)**
	**Healthexp**	**Healthexp**	**Healthexp**	**Healthexp**	**Healthexp**	**Healthexp**
Treatment	2,205.52^***^ (292.11)	2,004.90^***^ (286.72)	1,731.33^***^ (335.75)	1,442.01^***^ (392.02)	1,740.29^***^ (459.53)	2,062.57^**^ (870.04)
lnavgGDP		2,072.50^*^ (1,070.65)	119.10 (1,186.83)	1,014.31 (1,813.69)	−2,956.27^*^ (1,515.01)	
Lnpop		8,915.43^***^ (3,360.99)	6,531.09^*^ (3,815.37)		7,590.65 (5,879.76)	10,327.85 (9,281.13)
Lnsecindustry		702.98 (568.05)	2,450.51^***^ (738.22)	429.29 (828.57)	3,100.46^***^ (901.13)	−280.57 (396.90)
Capacity		−3,067.85^***^ (966.16)	−4,366.55^**^ (1,726.93)	−1,696.22 (1,202.73)		−591.29 (2,760.82)
Constant	1,147.30^***^ (126.16)	−58,112.40^***^ (15,362.77)	−51,274.07^***^ (17,545.79)	−11,695.55 (11,904.66)	−36,462.23 (24,510.04)	−33,773.82 (34,821.08)
Observations	1,640	1,640	1,281	663	909	559
R-squared	0.78	0.79	0.78	0.87	0.75	0.79
Number of county	237	237	190	94	177	154
r2_w	0.78	0.79	0.78	0.87	0.75	0.79

*Robust standard errors are in parentheses*.

*** *p < 0.01*,

** *p < 0.05*,

* *p < 0.1*.

*Columns (1) and (2) are the regression results after adding control variables. Column (1) only has a policy variable indicating the PMC reform dummy. Column (2) includes four variables of lnavgGDP, lnpop, lnsecindustry, and capacity, in which lnavgGDP is the per capita GDP (avgGDP) transformed to logarithm; and lnpop and lnsecindustry are the population and second industry output transformed to logarithm, respectively. Columns (3)–(6) are the results of hierarchical regression, where column (3) is the regression result of counties not including county-level cities; column (4) is the regression result of counties with pop >52.16; column (5) is the regression result of counties with capacity <0.30; and column (6) is the regression result of counties with avgGDP >13,426.70. All results are rounded up to two decimal places*.

## Discussion

The PMC reform of Jilin Province, Hebei Province, and Anhui Province had a significantly positive effect on the health expenditure of the county-level government, which is the important part of civilian public service expenditure. This result is consistent with the research results based on the county-level data of Henan Province from 2000 to 2013 that the productive and civilian public service expenditure of the counties directly managed by the province has been significantly increased (35). Another study on the impact of the PMC reform on per capita basic education expenditure reached the same conclusion (14). The PMC reform can significantly improve the fiscal expenditure and economic growth of counties and alleviate the financial difficulties of county-level governments, which will lay a solid economic and financial foundation for health expenditure. At the same time, the reform reduced the levels of local finance, which alleviated the phenomenon of a “city-scraping county” and increased the fiscal autonomy of local governments. The positive effects of the reform will encourage county-level governments to increase their health expenditure. Despite the positive effects above, the government health expenditure is still insufficient, so the government transfer payment system should be improved to ensure accessibility to basic health services in underdeveloped areas.

This study shows that the policy effect of the PMC reform on the proportion of health expenditure to fiscal expenditure of county-level governments is not significant. However, a study on 1,105 counties in the central and eastern provinces of China from 2002 to 2007 indicated that the PMC reform had a significant negative effect on the proportion of health expenditure to fiscal expenditure (32). A study on the impact of the PMC reform on the proportion of public education expenditure to the total expenditure in Henan Province also found a negative effect (35). The difference may result from the different samples used in these studies. Besides, according to the traditional decentralization theory, compared with the central government, local governments have the advantages of information and flexibility, and the provision of public services by local governments is more compatible with local residents' preferences and is more efficient (21, 36). People will respond by moving to a jurisdiction where the public provision level fits their preferences, but China has not only a household registration system that restricts the free movement of people but also a local tax system that is insensitive to the movement of people. Moreover, local governments do not have real tax legislative power in China. However, the potential benefits of decentralization also depend on the existence of decentralization of political decision-making authority, in particular effective channels for individuals to express their preferences and incentives for decision makers to respond to those preferences (37). For these reasons, many previous studies were skeptical of successful decentralization in developing countries. Obviously, these conditions do not exist in China at present. On contrary to other experiences in developing countries, political power has not been devolved yet in China: local government officials are not accountable to the local electorate but to higher-level government officials. In a word, China's fiscal federalism deviates considerably from the textbook case and thereby may yield quite different results.

Fiscal decentralization intensifies the competition among local governments and distorts their public expenditure structure, resulting in an increase in productive public service expenditure and a decrease in civilian public service expenditure (38). Fiscal decentralization can be divided into symmetric fiscal decentralization system and asymmetric fiscal decentralization system according to whether financial power and administrative power are symmetric. According to the proportion of fiscal revenue and fiscal expenditure between the central and local governments in China, fiscal decentralization in China is obviously asymmetric decentralization. The serious mismatch between financial power and administrative power also makes local governments more willing to spend their limited fiscal revenue on productive expenditure that is beneficial to them. It was also proven by the study results that the impact of asymmetric decentralization on health output in Papua Province was negative (39). Thus, only when the reforms of the administrative system and the fiscal system are carried out at the same time and perfect supervision mechanism is established, can the ideal reform effect be achieved. It is important to further improve the division of financial power and administrative power of the central and local governments at all levels. Besides, provincial governments need to strengthen the supervision and management of county-level governments and limit the irrational behavior of local governments with regard to fiscal expenditure.

The assessment of local officials focuses on indicators, such as GDP, fiscal revenue, and infrastructure (40, 41). As a result, local officials are more interested in repeated investment in infrastructure and other “achievement projects” during their term of office, instead of education, health, culture, and other public services, which ultimately lead to difficulties in the transformation of a local finance system from “constructive finance” to “public finance” (42). Therefore, whether the PMC reform has no effect or a significant negative effect, it reflects the following facts. To a certain extent, the reform gives local governments greater fiscal autonomy, improves the enthusiasm of county-level governments to develop the county's economy (43), and promotes improvement in county-level governments' health expenditure (44). However, the proportion of health expenditure to fiscal expenditure has not increased (32), and local governments lack the motivation to improve the supply of health services (45). Local governments still prefer a fiscal expenditure structure that emphasizes production over people's livelihood (31). In conclusion, the reform does not change the essence of local governments' political championship. Therefore, it is necessary to adjust the incentive mechanism of county-level governments and improve the promotion system for local government officials. It is suggested to include the evaluation indexes of public services, such as education and health, into the government performance evaluation system appropriately in order to gradually reverse the expenditure tendency of “emphasizing production, neglecting people's livelihood.”

Compared with previous studies on the influencing factors of government health expenditure from the perspective of macro-economy, the major contribution of this study is to study from the perspective of the fiscal system. What makes China's experience somewhat unique worldwide is the depth of fiscal decentralization on expenditure, in contrast to the recentralization of revenue since the tax-sharing system reform in 1994 (5). Comparing with previous studies on the impact of the PMC reform, we specifically studied the impact of the PMC reform on the health sector and focused on two indicators—health expenditure and its proportion to fiscal expenditure. We studied the impact of the PMC reform on not only the government expenditure but also the structure of government expenditure, which is more comprehensive. The three provinces had implemented the PMC reform earlier, so the duration is relatively long. Therefore, the research conclusion is more accurate considering the lag effect of reform.

However, this study has the following limitations. First, due to the limitation of data availability, this study only selected the three provinces of Jilin Province, Hebei Province, and Anhui Province as the research sample, which makes it difficult to represent the situation of the 24 provinces that are carrying out the PMC reform. Second, the fiscal expenditure data of counties in some years included in the study were not disclosed, and the missing proportion was 13.50%. In this study, cases containing missing values were excluded when the model was constructed, which weakens the reliability of the results. Third, the level of the aging population, changes in the spectrum of diseases of residents, and the implementation of the new rural cooperative medical care system in 2003 may have affected the level of health expenditure of county-level governments. However, due to the limited availability of data, this study did not incorporate them into the model as control variables. Therefore, future studies can increase the sample size, control other possible influencing factors, and further explore the effect of the PMC reform on county-level health expenditure.

## Conclusion

In this study, we evaluated the impact of the PMC decentralizing fiscal reform on health expenditure using longitudinal county-level data from 2003 to 2010 in Hebei Province, Anhui Province, and Jilin Province in China. Due to the different time points of reform, we use a time-varying DID method to estimate the effect of the PMC reform on health expenditure. We also controlled self-financing capacity, population, per capita GDP, and second industry output to account for any confounding effects on our estimates. The findings show that the fiscal system reform of the three provinces had a significantly positive effect on the health expenditure of county-level governments, whereas we found no effects on the proportion of health expenditure to fiscal expenditure of county-level governments. In order to change this situation, the government policy-making sectors need to perfect the supporting system while carrying out the PMC fiscal reform. There are four suggestions as follows: firstly, further specify the division of financial power and administrative power of the central and local governments at all levels; secondly, improve the government transfer payment system in health; thirdly, establish a scientific and rational government performance evaluation system and official promotion system; finally, strengthen the supervision and management of county-level government behaviors by provincial governments. These findings would serve as effective policy instruments aiming at achieving UHC by generating more additional resources for health sectors and minimizing the county-level disparity in the growth of health expenditure in China.

## Author Contributions

JZ contributed to the conception and design of the study. JH organized the database. CT performed the statistical analysis. JH, CT, JZ, and JJ wrote sections of the manuscript. All authors contributed to the article and approved the submitted version.

## Conflict of Interest

The authors declare that the research was conducted in the absence of any commercial or financial relationships that could be construed as a potential conflict of interest.
